# Oxidative Stress-Related Endothelial Damage in Vascular Depression and Vascular Cognitive Impairment: Beneficial Effects of Aerobic Physical Exercise

**DOI:** 10.1155/2019/8067045

**Published:** 2019-12-20

**Authors:** Maria Luca, Antonina Luca

**Affiliations:** Department of Medical, Surgical Sciences and Advanced Technologies “GF Ingrassia”, University of Catania, Italy

## Abstract

Oxidative stress- (OS-) related endothelial damage is involved in the occurrence and progression of several disorders, such as vascular depression and dementia. It has been reported that moderate, aerobic, physical exercise could reduce OS and inflammation, thus limiting the cardiovascular risk factors while improving endothelial homeostasis, mood, and cognition. In this review, we will discuss about the role of OS and OS-related endothelial damage in vascular depression and vascular cognitive impairment. Then, we will comment on the effects of physical exercise on both disorders.

## 1. Oxidative Stress, Endothelium, and Physical Exercise

During physical exercise, the production of reactive oxygen species (ROS), including hydroxyl radical (OH-), hydrogen peroxide (H_2_O_2_), and superoxide (O_2_-), increases. The fine balance between ROS and antioxidants, namely, redox homeostasis, is involved in various physiological processes including cellular signaling, gene expression, cellular growth, neurotransmitter release, and synaptic plasticity [1, 2]. Hence, a ROS overproduction can lead to detrimental effects for the cells. In fact, containing unpaired electrons, ROS are highly reactive molecules that can oxidize several cellular compounds, among which DNA, lipids, and proteins [3]. The main endogenous sources of ROS are probably the mitochondria, followed by peroxisomes and endoplasmic reticulum, while exogenous ROS-inducing agents are pollutants, xenobiotics, bacteria, radiations, diet, excessive physical exercise, etc. [4]. The imbalance between ROS generation and antioxidant substances determines oxidative stress (OS), condition favoring the occurrence and progression of several chronic illnesses such as dementia, movement disorders, depression, and cardiovascular disturbances [5–7]. In addition, ROS overproduction induces an inflammatory status, characterized by a release of cytokines, prostaglandins, and chemokines whose pivotal role in the occurrence of the previously mentioned disorders has been fully elucidated [8]. Among the conditions accompanied by sustained OS, overtraining has been demonstrated to determine an excessive production of ROS, thus favoring DNA damage, lipid peroxidation, and necrosis [9]. Conversely, moderate aerobic physical exercise leads to a transient elevated ROS production, an increased muscle contractility, an improvement of insulin sensibility, and a better regulation of vasodilatation [10]. Furthermore, moderate physical exercise improves the endogenous antioxidant defense system, stimulating the expression of superoxide dismutase 1, superoxide dismutase 2, glutathione peroxidase, and glutathione reductase, while reducing the concentration of several inflammatory markers, including interleukin- (IL-) 6, homocysteine, and tumor necrosis factor-alpha [11]. From a clinical point of view, the beneficial effects of moderate physical exercise result in a lower incidence of ischemic stroke, heart attacks [12], obesity, and diabetes [11] among trained individuals. Exercise seems to exert its beneficial role both directly, improving the insulin sensitivity and the mobilization of fatty acids and glucose, and indirectly, by favoring weight loss [13]. Moreover, a recent animal study has demonstrated that exercise increases the endothelial nitric oxide synthase (eNOS) expression and activation both in the vascular wall and in the perivascular adipose tissue, thus restoring their anticontractile capacity with the consequent improvement of the vascular function [14]. The latter mechanism could explain, at least in part, the beneficial role of exercise training on several acute and chronic disorders related to vascular dysfunction, including vascular cognitive impairment (VCI) and vascular depression (VaD). In this review, we will discuss about the role of OS and OS-related endothelial damage in VCI and VaD. Then, we will comment on the effects of physical exercise on both disorders.

## 2. The Role of Vascular Endothelium in Depression

### 2.1. Vascular Depression

Depression is one of the most frequent mental disorders (lifetime prevalence of 20.6%) that, according to the World Health Organization projections, could become the leading cause of disability by 2030 [15, 16].

The most frequent depressive symptoms are sad mood, thoughts of guilt, lack of interest, sleep disorders, lack of appetite, psychomotor retardation or agitation, cognitive deficits, and suicidal ideation [17, 18]. Among the different neurobiological mechanisms contributing to the occurrence of depression, it is possible to include the serotoninergic, norepinephrinergic, and dopaminergic deficits, the dysfunction of the hypothalamic-pituitary-adrenal (HPA) axis, the hyperproduction of proinflammatory mediators, and an altered gut-brain axis [19]. Despite the median age at depression onset typically being the early adulthood [20], depressive symptoms are common in the elderly and are associated with reduced quality of life and elevated mortality [21]. Over the years, several factors have been variably associated with the occurrence of late-life depression, including social isolation, poor family support, low education, marital status [22], Parkinson's disease, diabetes, cancer, and cerebrovascular disorders [22–24]. The association between cerebrovascular disorders and depression in late-life has been so frequently reported that the concept of “*vascular depression*,” subtype of late-life depression frequently predisposed or perpetuated by an ischemic damage of frontosubcortical circuits, emerged [25]. As a matter of fact, a recent meta-analysis supported the association between brain silent lesions due to vascular damage (white matter lesions (WMLs)) and late-life depression [26]. WMLs are commonly observed in the elderly; they are frequently related to structural and functional cerebrovascular pathology and are facilitated by the endothelial dysfunction [27–29].

### 2.2. Vascular Endothelium and Vascular Depression

Vascular endothelium is a single cellular layer that lines the walls of all blood vessels. Releasing both vasodilators (i.e., nitric oxide) and vasoconstrictors (i.e., endothelin), it regulates the vasomotor tone, thus exerting a fundamental role in the prevention of cerebrocardiovascular disorders [30]. Endothelial dysfunction could contribute to the occurrence of VaD, as discussed below.

Firstly, an association between risk factors for endothelial damage (i.e., hyperglycemia, hypertension, and dyslipidemia) and depression has been reported [31, 32]. Moreover, homocysteine, a toxic amino acid derived from dietary methionine and representing a strong risk factor for cerebrocardiovascular disorders (due to vasodilatation impairment), has been found to be elevated in depressed patients [33]. Interestingly, depression is frequently characterized by functional alterations affecting crucial components of the blood-brain barrier (BBB), such as a reduced E-cadherin expression (resulting in less efficient tight junctions) and an impaired function of claudin, a protein contributing to the formation and maintenance of the BBB. These functional changes are favored by the prooxidative and proinflammatory status [34]. In fact, OS seems to directly upregulate the endothelial expression of NMDA receptor in the neurovascular unit, thus mediating endothelial excitotoxicity [35]. Another OS-related condition characterizing depression is the activation of metalloproteinases 2 and 9, proteins demonstrated to increase the BBB permeability and stimulate the inflammatory status [34, 36, 37]. A dysregulated function of metalloproteinases, altering the neurovascular unit, favors neuronal damage and cognitive impairment. Consistently, high levels of metalloproteinase 9 have been detected in the vascular walls, senile plaques, and neurons of brains affected by Alzheimer's disease (AD) [38]. Interestingly, physical exercise has been repeatedly shown to reduce the levels of metalloproteinases [39, 40] and to improve the endothelial function, also through its stimulatory effect on eNOS [41, 42].

The since here discussed molecular alterations could account, at least in part, for the frequent association between depression and neurological conditions (i.e., traumatic brain injury, stroke, and multiple sclerosis) characterized by endothelial dysfunction and inflammation [34]. Concerning the latter, depression is characterized by the release of inflammatory molecules inducing vascular damage, such as IL-6- and cytokine-like leptin, an anorexogenic agent linked to the eating disturbances frequently associated with late-life depression [43–45]. In the latter type of depression, the endothelial damage is further stimulated by the age-related dysfunctional hyperactivation of glial cells, determining the release of several proinflammatory cytokines and ROS hyperproduction [46, 47]. Finally, the autonomic dysfunction characterizing mood disorders may as well sustain endothelial dysfunction [48, 49]. For example, an imbalance between sympathetic and parasympathetic tone, common in the elderly, has been related to myocardial infarction and ischemic stroke [30]. As a matter of fact, the relationship between vascular damage and depression seems to be “bidirectional”: if, on the one hand, endothelial damage could sustain depression, on the other hand, depression itself represents a cerebrocardiovascular risk factor. As a result, elderly depressed patients frequently show an impairment in activities of daily living as well as cognitive decline and increased medical comorbidity [50].

## 3. Vascular Cognitive Impairment and Endothelium

The term *vascular dementia*, recently substituted by the more inclusive term “*vascular cognitive impairment*” (VCI), encompasses a range of cognitive disorders strictly related to vascular disease [51]. Despite the large variability in the esteems, VCI is still considered the second most common type of dementia, after AD, accounting for 15-20% of individuals affected by dementia [52]. Epidemiological studies reported that cerebrovascular disorders increase from 3.5 to 47 times the risk of dementia, depending on the severity of the vascular event (i.e., silent WMLs, transient ischemic attacks, minor stroke, and stroke) [53]. The differences in the epidemiological data can be reconducted in the variety in terms of frequency, features, and prognosis characterizing the disorders classified under the umbrella term “VCI.” It should be noted, in fact, that the many faces of VCI reflect the heterogeneous nature of the vascular damage and its distinct contribution to the occurrence of cognitive decline. Hence, among VCI disorders, it is possible to include multi-infarct (cortical vascular) dementia, small vessel (subcortical vascular) dementia, strategic infarct dementia, hypoperfusion dementia, hemorrhagic dementia, hereditary vascular dementia (including the cerebral autosomal dominant arteriopathy with subcortical infarcts and leukoencephalopathy CADASIL), and AD with cerebrovascular disease (cooccurrence of neurodegeneration and vascular damage) [54]. Cerebral small vessel disease is the most common form of brain vascular disorder [55] and is held responsible not only for vascular dementia but also for vascular parkinsonism, sometimes difficult to distinguish from idiopathic Parkinson's disease and atypical parkinsonism [56, 57].

Vascular brain pathology is anything but rare in the elderly and could cause cognitive deficit ranging from mild cognitive impairment to dementia [58]. From a clinical point of view, VCI is characterized by extremely variable cognitive deficits, highly dependent on vascular damage localization and severity, as previously stated. However, due to the frequency of small vessel disease, clinical manifestations related to the frontostriatal circuit impairment, including executive function and attention deficits, are usually observed [59]. The most studied risk factors variably associated with VCI are represented by old age, low educational level, smoking status, hypertension, diabetes, obesity, and hypercholesterolemia [60]. These factors, some of them modifiable, cooccur in a scenario of age-related modifications exerting a detrimental effect on the endothelium. In fact, from a molecular point of view, aging is characterized by an imbalance between oxidants and antioxidants. The consequent ROS overproduction in the vascular system, including brain circulation, could be partially related to the overexpression of NADPH oxidases, characterizing the elderly [61]. In addition, OS determines the hyperactivation of glycogen synthase kinase (GSK-3) and, consequently, an alteration of mitochondrial permeability, recognized to exert a pivotal role in the pathogenesis of atherosclerosis [62, 63]. Moreover, aging is characterized by microglial hyperactivation. This condition represents a defensive response to inflammation; however, particularly when sustained, it constitutes a dangerous source of free radicals, ultimately favoring OS and neurodegeneration [50]. The latter could be further facilitated by the aging-related impairment of the brain autoregulation myogenic tone and the BBB breakdown, leading to the passage of immune cells and toxins into the brain [64, 65]. On the light of what has been said, the interplay between inflammation and OS could represent the biological basis of endothelial damage and BBB break failure occurring in VaD [66, 67].

## 4. Physical Exercise in Vascular Depression and Vascular Cognitive Impairment

The pharmacological treatment of depression and dementia in the elderly is frequently complicated by poor compliance, drug side effects, and interactions due to multiple therapies. Therefore, nonpharmacological approaches, as monotherapy or in add-on to pharmacological agents, are receiving increasing interest.

Physical exercise exerts beneficial effects in terms of psychological well-being, quality of life, and depressive symptoms [68]. In fact, it has been demonstrated to be as effective as the antidepressant sertraline and to reduce depressive symptoms in nonresponders to antidepressants [69, 70]. Notably, a relatively recent meta-analysis confirmed the benefit of exercise on depression, even in comparison with active usual care (i.e., social contact) [71]. Since vascular dysfunctions are shared by both late-life depression and VCI [72], VaD could be more than a risk factor for VCI. In fact, a continuum from late-life depression to VCI could be hypothesized. In this context, physical exercise may represent a viable strategy to prevent cognitive impairment in the elderly. In fact, studies carried out on middle-aged individuals with normal cognition identified moderate cardiorespiratory fitness as a protective factor against cognitive impairment, being able to improve attention and executive function performances [73, 74]. These findings could find an explanation in some molecular mechanisms highlighted in humans and animal models of AD. For instance, physical exercise seems to improve the clearance of A*β* plaques and hyperphosphorylated tau protein [75]. On the other hand, physical exercise limits OS, favoring an increase of the peripheral insulin-like growth factor 1, stimulating the activity of superoxide dismutase and decreasing the NADH oxidase activity [76]. A further mechanism supporting the beneficial role of physical exercise in VCI is represented by the stimulation of the neurogenesis in the subventricular and subgranular zone and an increased angiogenesis of capillaries from existing vessels [76]. Interestingly, randomized controlled trials have confirmed that physical exercise may delay the cognitive decline characterizing AD [77] and VCI [78, 79]. Unfortunately, despite the recommendations of the World Health Organization and the encouraging evidence, physical inactivity is still a major concern when considering middle-aged individuals [80]. Future studies are needed in order to strengthen the current evidence regarding the role of physical exercise in both VaD and VCI and raise awareness on this issue.

## 5. Conclusion

The previously discussed evidence highlights the beneficial effects of moderate, aerobic, physical exercise in reducing the cerebrocardiovascular risk factors, as well as improving endothelial homeostasis, mood, and cognition. Hence, the elderly should be encouraged to maintain an active lifestyle to reduce the risk of disabilities in late-life.
